# Supplementary figures and images for: Safety assessment of Mpp75Aa1.1, a new ETX_MTX2 protein from Brevibacillus laterosporus that controls western corn rootworm
Source: PLoS One. 2022 Sep 8;17(9):e0274204. doi: 10.1371/journal.pone.0274204 (PMC9455866; doi:10.1371/journal.pone.0274204)

Fig 1

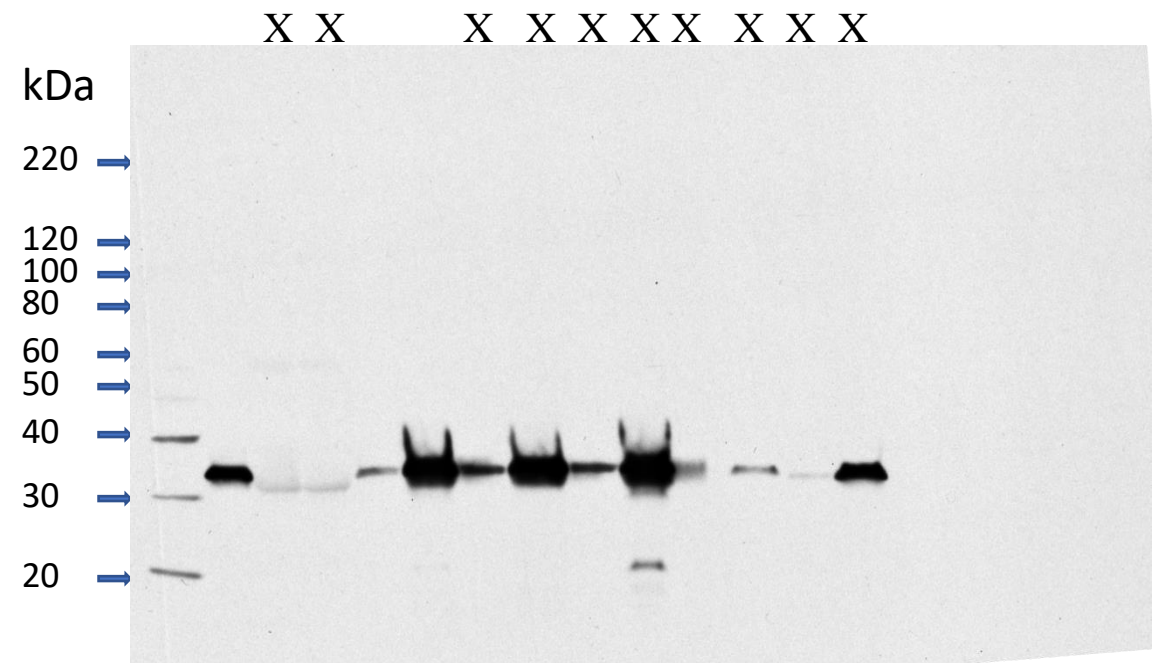

Fig 2

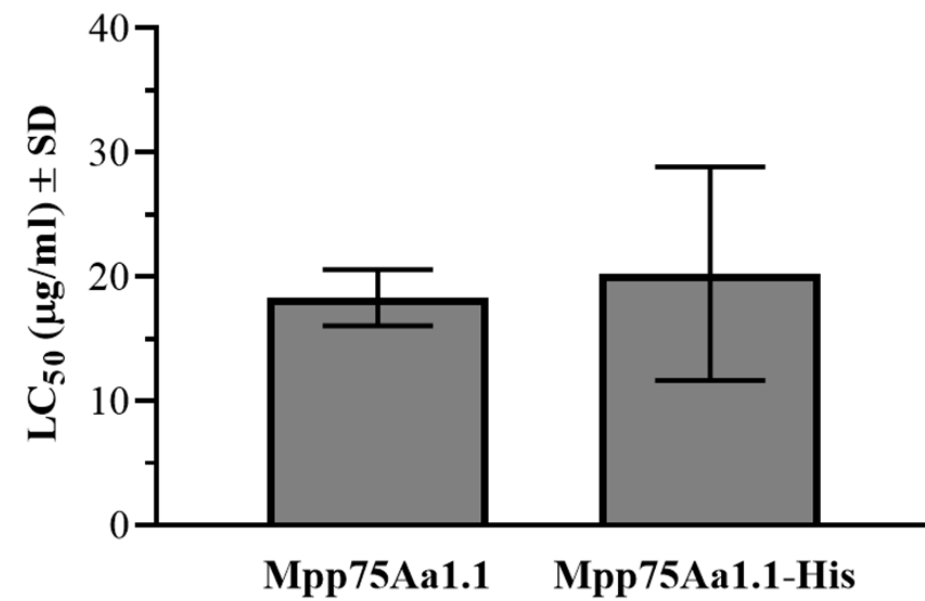

Fig 3A

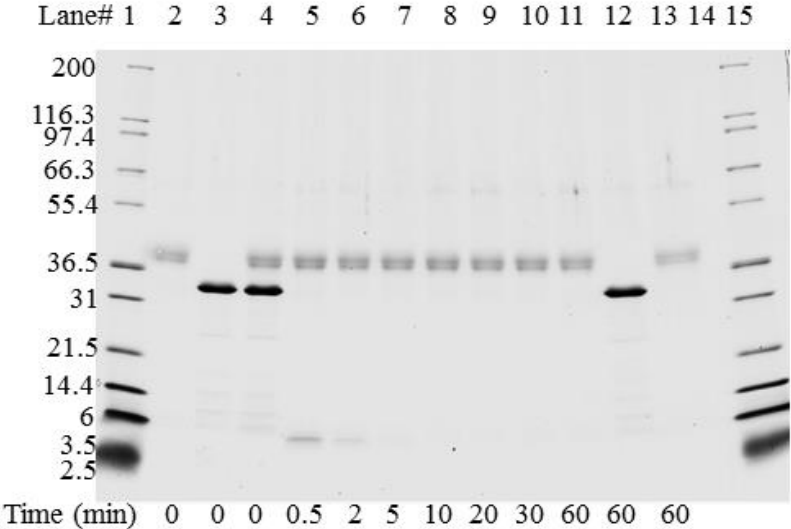

Fig 3B

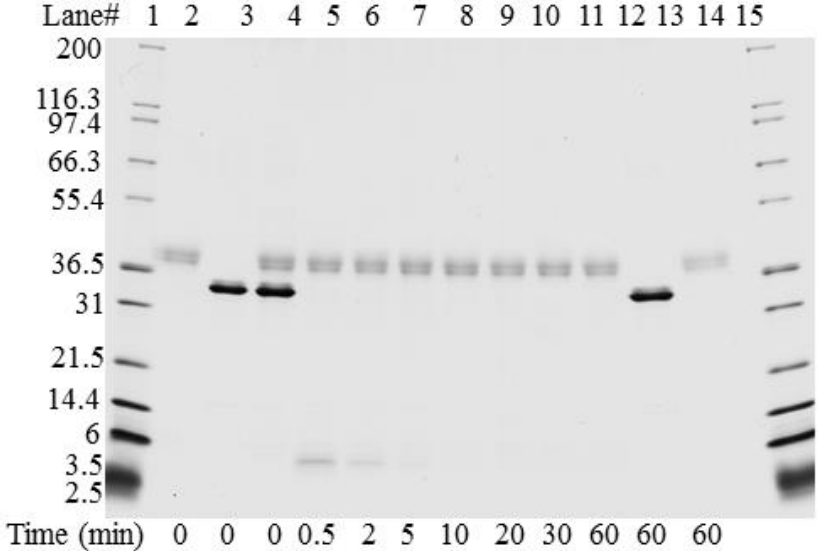

Fig 4

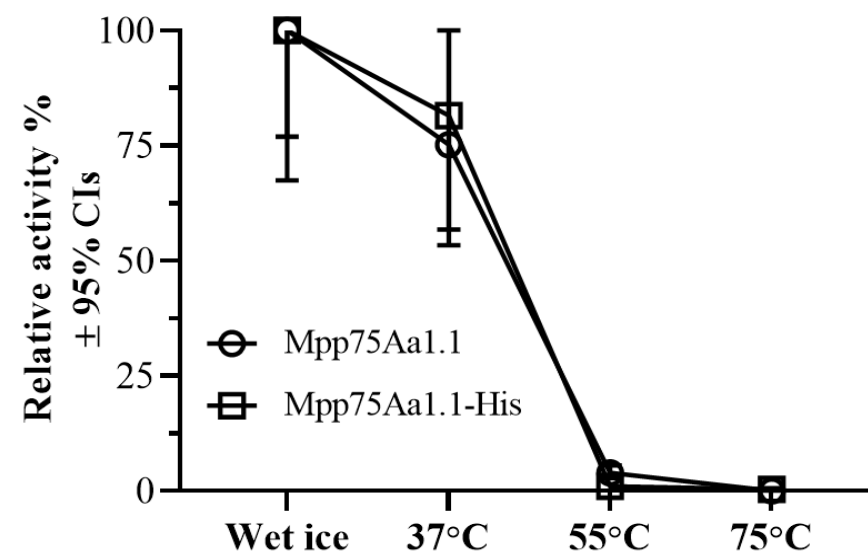

Supplement: S1 Raw images — (PDF) [file pone.0274204.s001.pdf]
