# Supplementary material for: Safety assessment of Mpp75Aa1.1, a new ETX_MTX2 protein from Brevibacillus laterosporus that controls western corn rootworm
Source: PLoS One. 2022 Sep 8;17(9):e0274204. doi: 10.1371/journal.pone.0274204 (PMC9455866; doi:10.1371/journal.pone.0274204)
Supplement: S1 File — (PDF) [file pone.0274204.s002.pdf]

Supplemental 1

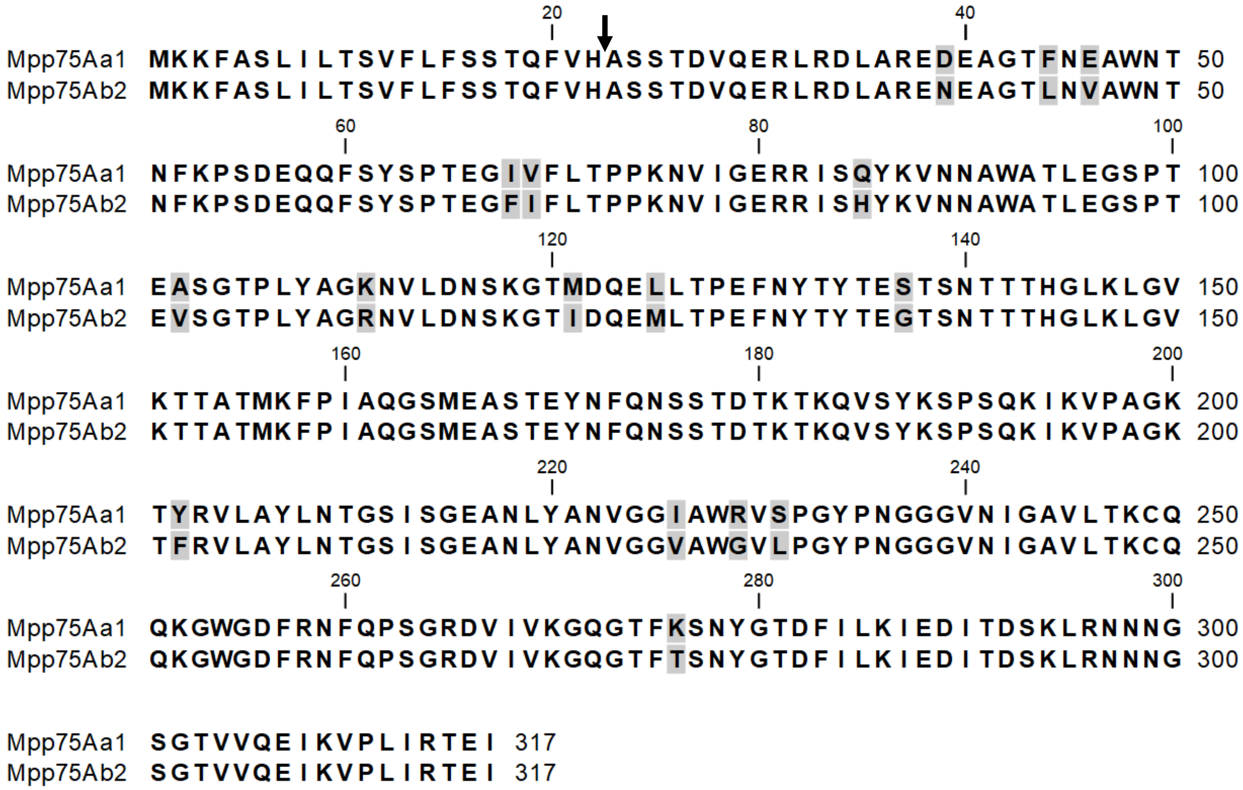

S1. Multiple sequence alignment of the amino acid sequence of Mpp75Aa and Mpp75Ab. A multiple sequence alignment of Mpp75Aa and Mpp75Ab was built, using Clustal W, with the full-length amino acid sequences. The arrow between amino acids 23 and 24 shows the site where the N-terminal membrane transiting signal peptide is cut and removed, and the start of the mature form of the protein. The gray boxes highlight amino acids that differ from the consensus.

Supplemental 2

		1	2
Mpp75Aa1	1		16
Mpp75Ab2	2	94.95	

S2. A pairwise comparison of the Mpp75Aa and Mpp75Ab homologs. The number in the lower left box is the percent identity between the sequences and the upper right is the number of amino acid differences between the pair.

Supplemental 3

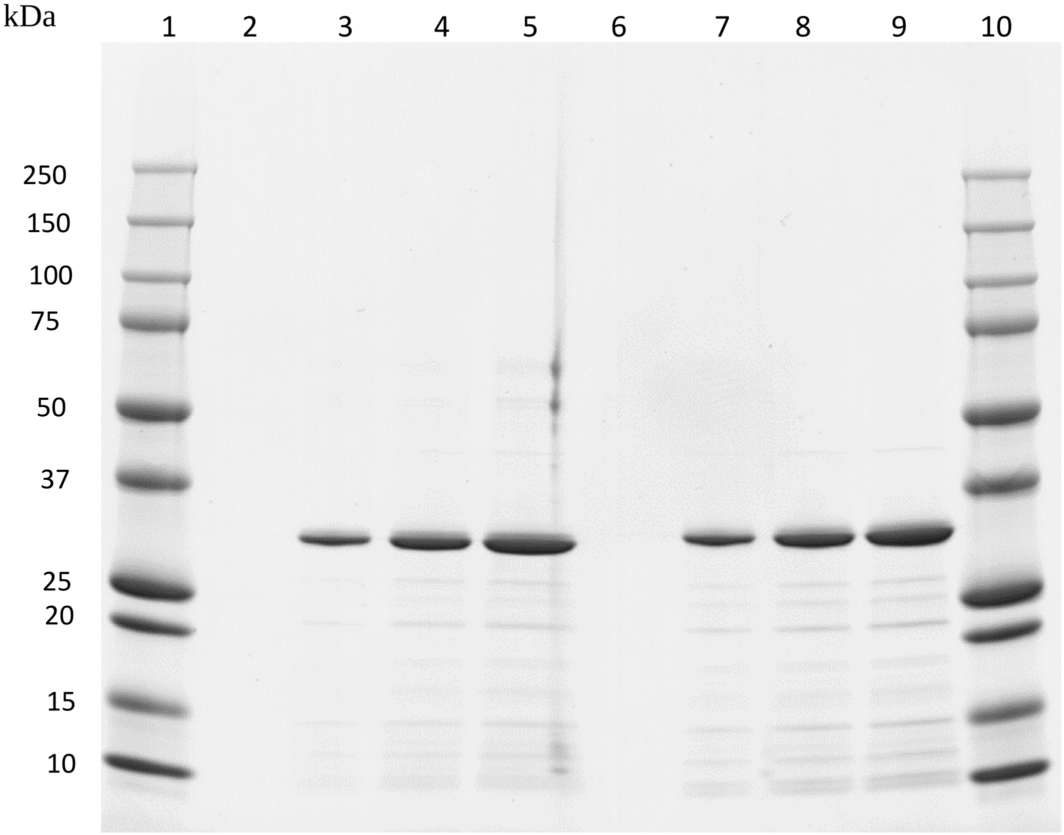

S3: SDS-PAGE analysis of purified Mpp75Aa1.1 proteins

Protein samples were subjected to pre-cast Tris-glycine 4-20% (w/v) SDSPAGE and stained with Brilliant Blue G-Colloidal stain. Molecular weight markers (kDa) are shown on lanes 1 & 10; lanes 2 & 6: empty; lanes 3 to 5: Mpp75Aa1.1; lanes 7 to 9: Mpp75Aa1.1-His; lanes 3 & 7: 1 μg lanes 4 & 8: 2 μg; lanes 5 & 9: 3 μg.
